# Dalbavancin in the Treatment of Bacteremia and Endocarditis in People with Barriers to Standard Care

**DOI:** 10.3390/antibiotics9100700

**Published:** 2020-10-15

**Authors:** Leama Ajaka, Emily Heil, Sarah Schmalzle

**Affiliations:** 1 Department of Medicine, University of Maryland Medical Center, Baltimore, MD 21201, USA; sschmalzle@ihv.umaryland.edu; 2 Department of Pharmacy Practice and Science, University of Maryland School or Pharmacy, Baltimore, MD 21201, USA; eheil@rx.umaryland.edu; 3 Department of Medicine, Division of Infectious Disease, University of Maryland School of Medicine, Baltimore, MD 21201, USA; 4 Institute of Human Virology, University of Maryland School of Medicine, Baltimore, MD 21201, USA

**Keywords:** dalbavancin, infective endocarditis, injection drug use, substance use disorder, people who inject drugs

## Abstract

Introduction: Dalbavancin is an antibiotic administered by intravenous infusion weekly or bi-weekly and is currently FDA-approved only for treatment of skin and soft-tissue infections. It has shown promise, but is not considered the standard of care, for bacteremia and infective endocarditis (IE), which typically require outpatient parenteral antibiotic therapy (OPAT) for prolonged durations. People who inject drugs (PWID) with bacteremia or IE are often perceived as having barriers to OPAT and standard daily-administered antibiotics, prompting off-label use of dalbavancin in this population. Methods: A retrospective review of adult patients receiving at least one dose of dalbavancin for bacteremia or IE was conducted between 1 November 2017 and 31 October 2019. Outcomes and reasons for use of dalbavancin were recorded, including specific barriers to standard therapy. Results: Stated reasons for dalbavancin use in the 18 patients identified included active injection drug use (50%), inability to arrange standard OPAT due to patient adherence or inability to place in skilled nursing facility (SNF) (22%), risk for additional infections or other morbidity with OPAT (22%), and patient preference (6%). In 11 patients (61%) SNF placement was not attempted due to behavioral issues or patient declination. There were five patients who did not complete their intended course of treatment (28%). At 90 days, eight patients (44%) achieved a clinical or biologic cure, six (33%) failed treatment, and four (22%) were lost to follow-up. Conclusion: Dalbavancin may have a role as salvage therapy in the treatment of IE and bacteremia in PWID who have significant barriers to standard treatment.

## 1. Introduction

Dalbavancin is a long-acting intravenous (IV) antibiotic active against Gram-positive bacteria, including methicillin-resistant *Staphylococcus aureus* (MRSA). It is a synthetic glycopeptide that has the same mechanism of action as vancomycin; it binds to the terminal d-alanyl-d-alanine of growing peptidoglycan chains and inhibits bacterial cell wall synthesis. It has more potent in vitro bactericidal activity against Gram-positive organisms than vancomycin, possibly because dalbavancin has a novel ability to anchor its lipophilic side chain in the bacterial membrane. In healthy adults with normal renal and hepatic function, it exhibits linear and dose-dependent pharmacokinetics. Dalbavancin has a half-life of 170–210 hours following administration of one IV dose [1]. Its long half-life allows for dosing every 1 or 2 weeks [2,3]. Furthermore, it is well-tolerated, with analyses of randomized controlled trials showing adverse event rate similar or lower than that of comparable agents [4]. 

Dalbavancin is currently FDA-approved for the treatment of skin and soft-tissue infections. Treatment courses for skin and soft-tissue infections typically require antibiotics ranging from 5 to 14 days, meaning one dose of dalbavancin is sufficient [5,6]. It is likely that dalbavancin is also effective in more serious infections. Case reports and randomized clinical trials have demonstrated success in treating more deep-seated or morbid infections, such as pneumonia, osteomyelitis, and endovascular infections, which typically require extended antibiotic durations; however, dalbavancin is not FDA-approved for these indications and is not yet considered the standard of care for these sites [7,8,9,10,11]. There are also data suggesting that dalbavancin can be an effective treatment for bacteremia and infectious endocarditis (IE) [12,13,14,15,16,17]. First-line treatment for bacteremia and IE includes the use of parenteral antibiotics for 2–6 weeks, depending on pathogen and patient characteristics [18]. This requires long-term venous access, such as a peripherally inserted central catheter (PICC), and outpatient parenteral antibiotic therapy (OPAT), either in the patient’s home or a skilled nursing facility (SNF). For a subset of patients, these first-line treatments are not possible due to social and health system barriers in arranging standard antibiotic courses. This includes people who inject drugs (PWID), those with repeated hospital discharges against medical advice (AMA), and patients with behavioral issues, such as elopement from medical facilities, inpatient drug use, or threatening behavior [14]. For some of these patients in our center, dalbavancin has been used in lieu of standard therapy for treatment of bacteremia or IE. With only limited data available on the use of dalbavancin for this indication, there are no defined standard dosing or interval recommendations and there are likely variations in provider practice [19].

Herein, we describe a series of patients receiving off-label dalbavancin for bacteremia or IE, the majority of which were PWID or with other substance use history.

## 2. Methods

### 2.1. Study Location, Design, and Eligibility

This is a retrospective observational study conducted at the University of Maryland Medical Center (UMMC), a 750 bed acute tertiary care center in Baltimore, MD. A retrospective chart review was conducted to identify cases from December 2017 to June 2019, in which adult patients received at least 1 dose of dalbavancin in the treatment of bacteremia or IE. During this study period, all dalbavancin prescriptions were made at the clinical discretion of the infectious diseases (ID) physicians evaluating the patient. 

### 2.2. Data Extraction and Definitions

Charts were each reviewed by an internal medicine resident, ID physician, and ID pharmacist. Charts were abstracted for relevant patient demographics, including age, gender, and race. Presence of active injection drug use or other substance abuse (drug use by any route, or alcohol abuse) within 30 days was also noted. ID notes and microbiology data were examined to determine infection being treated, and cases of bacteremia or IE were included in this analysis. Organisms and likely source of infection were identified, as well as if source control was obtained (if relevant). For patients receiving treatment for IE, cases were reviewed to determine if modified Duke criteria were satisfied by either clinical or pathologic criteria [20]. Also documented were any antibiotic treatments received prior to dalbavancin for the relevant infection, as well as the date of the patient’s last negative blood cultures in relation to when dalbavancin was received. Reasons for a non-standard antibiotic course were identified through primary team, ID consultant, case manager, and social worker documentation. These reasons were categorized as active injection drug use, post-hospitalization patient placement or patient adherence issues, general infection risk or morbidity with standard therapy, and patient preference. Dalbavancin treatment courses were identified, including dates, doses, and intended and completed numbers of doses. Also abstracted were elements of the hospital stay, including suspected drug use in the hospital, length of stay, denial from SNFs, number of days of clinical stability while awaiting SNF placement, and type of discharge (i.e., regular discharge, AMA discharge, or elopement). 

Clinical outcomes were classified as cure, failure, or loss to follow-up (LTFU). Cure was defined as lack of evidence of clinical or microbiological persistent or recurrent infection within 90 days or negative blood cultures within 90 days after completion of dalbavancin. Those categorized as failure experienced clinical or microbiologic relapse within 90 days (signs of infection, positive culture data, or infection-related death). LTFU was defined as any patient not having a subsequent encounter to evaluate their infection at either UMMC or any institution linked via the electronic medical record within 90 days. Outcomes were adjudicated and agreed upon by all three study team members.

### 2.3. Data Analysis

Descriptive statistics were performed for all data using Microsoft Excel software. 

The study was approved with a waiver of informed consent by the University of Maryland, Baltimore Institutional Review Board.

## 3. Results

Dalbavancin was used a total of 60 times from December 2017 to June 2019 at UMMC, but in only 18 cases (*n* = 18) for bacteremia or IE. Of these cases, the majority of dalbavancin use was for bacteremia (78%). In four cases (22%), dalbavancin was used for treatment of IE. Of cases in which it was used for endocarditis, two were cases of definitive IE and two were possible IE (following modified Duke criteria). There was one patient who was presumed to have bacteremia given overwhelming evidence of disseminated MRSA infection, but did not have any positive blood cultures. MRSA and methicillin-susceptible *Staphylococcus aureus* (MSSA) were the causative organisms in seven (39%) and three (17%) cases, respectively. In three cases (17%), dalbavancin was used in the treatment of polymicrobial Gram-positive organism and Gram-negative rod infection. In two cases (11%) dalbavancin was used in the treatment of polymicrobial infection with multiple Gram-positive organisms. One patient received dalbavancin for the treatment of group A streptococcus infection, one for *Streptococcus mitis*, and one for *Staphylococcus lugdunensis* infection.

In half (50%) of the cases, the source of bacteremia or IE was unknown. In four cases (22%), the source of bacteremia or IE was likely skin and soft-tissue infections (3 abscesses; 1 tenosynovitis). In two cases (11%), the source of infection was thought to be osteomyelitis. One patient developed bacteremia from pneumonia, which was complicated by empyema formation, and one from an indwelling catheter that was present prior to admission. Of patients with a presumed source, whether it was empyema, abscess, osteomyelitis, or an indwelling catheter, all but two had source control of their infection (including incision and drainage, amputation, or chest tube and decortication). One patient each with pelvic osteomyelitis and tenosynovitis were treated medically only, with no surgical intervention. All patients had documented negative blood cultures prior to receiving dalbavancin except for two—one patient who had left against medical advice and one patient who actually never had positive blood cultures, but instead had evidence of disseminated MRSA infection, and thus was presumed to have bacteremia. In all cases, the patient had received at least one antibiotic prior to initiating treatment with dalbavancin; in sixteen cases (89%), that antibiotic was vancomycin. Patients had received between 2 and 30 days (average 12.3 days) of other antibiotic therapy before therapy was changed to dalbavancin. 

The majority of patients that received dalbavancin for bacteremia or IE were PWID (67%), while one patient (5.5%) was not classified as PWID but had other active substance abuse issues. Of the PWID, 92% also abused other substances such as cocaine or alcohol. Six of 18 patients (33%) were suspected of using illicit drugs while hospitalized. The majority were deemed to be poor candidates for or declined PICC. Stated reasons for dalbavancin use were active injection drug use (50%), issues with SNF placement or patient adherence (22%), concern for line manipulation or infection, and patient preference (6%). No patients were considered appropriate candidates for home OPAT. In 61% of cases, SNF placement was not attempted due to behavioral issues or patient declining any SNF placement. Behavioral issues identified included prior elopement from SNF; continually leaving the medical ward with PICC in place, thus raising suspicion for PICC manipulation for drug use outside of hospital; suspected drug use in the hospital; or threatening hospital staff. In three cases, the patient was denied from at least one SNF and declined the alternative placement options. One patient was denied from every possible SNF in the region. Three patients left against medical advice (AMA) or eloped prior to completion of treatment for bacteremia or IE. These patients either received dalbavancin immediately before leaving the hospital due to planned AMA discharge or elopement or during outpatient follow-up. The median hospital stay was 14 days (range: 4–33 days), with an average of 4 days of clinical stability while awaiting SNF placement (range: 0–13 days). In 3 cases, patients were clinically stable for 10 or more days while attempts at SNF placement were made. 

The intended number of doses was one to three for all patients (average: 2), with patients completing one or two doses (average: 1). Five (28%) did not complete the intended course. Thirteen patients received 1.5 g as a single dose. Two patients received 1.5 g followed by another 1.5 g 7–14 days later. Two patients received 1.5 g followed by 1 g 7–14 days later. One patient received 1 g followed by 500 mg 8 days later. Eight patients achieved clinical cure (45%), six failed therapy (33%), and four were LTFU (22%). Eight patients were readmitted (related or unrelated to initial complaint) within 90 days of discharge. Three patients died within one month of receiving dalbavancin; one was thought to have died from complications of IE. One patient died of fulminant *Clostridium difficile* colitis and another died from presumed narcotic overdose. However, as it is unknown if the initial disease state for which they received dalbavancin or the dalbavancin treatment ultimately contributed to cause of death, all three of these outcomes have been labeled failures. 

Of note, two patients who received incomplete courses of dalbavancin grew a strain of MRSA with an elevated minimum inhibitory concentration (MIC) to vancomycin on a subsequent admission (MIC of <0.5 increased to 1 in both).

The patient demographics, infection details, treatment, and hospital course for each patient are included in Table 1.

## 4. Discussion

This review demonstrates a potential role for dalbavancin in the treatment of bacteremia and IE in patients with substance abuse disorders and barriers to standard therapy. The current modus operandi typically does not recommend that patients with substance abuse disorder be offered home OPAT [21]. The appropriateness for home OPAT in PWID is an area of active debate. There are examples of success with home OPAT in this population, but many physicians remain uncomfortable with PICCs and home OPAT in PWID [22]. If a patient has demonstrated a pattern of non-adherence and injurious behavior, PICC and even SNF placement may be viewed as unacceptable options given the risk of treatment failure and further morbidity and mortality. In patients with ongoing drug use, new bloodstream infections are common when receiving parenteral treatment for infective endocarditis [23]. While carefully selected PWID can be considered for OPAT at home, there is still a tremendous need to identify alternative treatment strategies for PWID with endovascular infections. Even in PWID who are motivated to adhere to OPAT, there can be concomitant issues that serve as roadblocks to safe and consistent antibiotic delivery, such as housing instability and lack of transportation or support systems [24]. Although newer data indicate that oral therapy could be utilized for partial treatment of endocarditis, non-adherence to the regimens could be a potential barrier to treatment success, particularly medications dosed multiple times per day [25]. In the 2019 Partial Oral versus Intravenous Antibiotic Treatment of Endocarditis (POET) study, the oral antibiotic regimens studied were often a combination of multiple antibiotics dosed twice or even four times daily. Furthermore, only a very small number of PWID were included and history of non-adherence was an exclusion criterion [26]. There have been studies showing that adherence to antibiotic therapy after discharge is low, even in general populations, and is unsurprisingly associated with poor clinical response. Furthermore, it is common for patients to overestimate or overstate their medication adherence, which increases the challenges in identifying those at risk for treatment failure [27]. The structural vulnerabilities (such as home insecurity or lack of a support system) that make OPAT difficult to arrange with PWID also affect adherence to both short- and long-term oral antibiotic treatment. Therefore, long-acting antimicrobials such as dalbavancin still present a very appealing niche for treatment of bacteremia and IE in PWID.

Additionally, even partial treatment may be better than none; one study in PWID leaving AMA showed that receipt of sub-par oral antimicrobials for invasive infections resulted in lower readmission rates [28]. Furthermore, dalbavancin confers the probable benefit of directly observed therapy, which is generally not possible for outpatient oral microbial regimens. However, while dalbavancin is a promising new therapy option, the risks of incomplete treatment do exist. In this series, two patients have grown MRSA with increased MIC to vancomycin following incomplete treatment courses with dalbavancin. This finding of increasing MIC is not entirely unexpected, given the similar mechanism of action between the antibiotics [29]. The elevated MICs to vancomycin remained within the susceptible range, but it is foreseeable that resistance could occur with continued sub-optimal treatment courses given the prolonged half-life of the drug [16,30]. Pairing use of dalbavancin with transitions-of-care programs that provide intensive patient support could assist in preventing loss to follow-up for future dalbavancin doses and clinic visits. Transitions-of-care programs that focus on medication management and adherence, potentially utilizing ambulatory care pharmacists or pharmacy technicians, may be a sustainable and cost effective way to ensure appropriate medicine education and follow-up [31]. The lay healthcare worker model, in which a member of the community who has received training to promote or carry out healthcare services, has also been implemented to assist high-risk patients with post-discharge social needs and has been shown to significantly reduce 30 day hospital readmission rates [32]. Interdisciplinary care and communication between hospitals, pharmacists, ambulatory providers, social workers, and mental health professionals is necessary in treating patients with complex infections that have barriers to healthcare. These methods may be a way to get vulnerable patients through a complete treatment course of dalbavancin and improve outcomes. It is also possible for home infusion companies to administer dalbavancin at home or at an SNF for patients who have difficulty with follow-up in clinics. 

Recent evidence has shown that long-term outcomes following IE in PWID are dismal. In one study in the United Kingdom that followed PWID during and after episodes of IE, 56% of patients were dead in 10 years. The cause of death was most often another infection (mostly IE) [33]. Treating patients with dalbavancin for IE and bacteremia may be a worthwhile harm reduction effort to improve long-term outcomes. Furthermore, the use of dalbavancin in skin and soft-tissue infections has been associated with decreased number of hospitalizations and decreased hospital length of stay. One report, which analyzed clinical outcomes of administering dalbavancin 7–10 days prior to the end date of parenteral therapy, found that in 81% of cases, this approach appeared to lead to successful treatment of the infection. Furthermore, it averted approximately 7 in-hospital days per patient, as well as saved an average of $9600 per patient [34]. This was certainly a motivating factor in its use in our institution—to facilitate discharge in patients who posed a risk to hospital staff or themselves should they remain in the hospital, and especially in those who wished to be discharged against medical advice. Patients in our study were clinically stable for an average of 4 days prior to discharge (and up to 13 days), demonstrating that delays in discharge are frequent and can be quite prolonged when attempting to set up parenteral antibiotics and SNF placement. It is reasonable to assume that the use of dalbavancin in bacteremia and IE could help eliminate these delays, and thus reduce hospital stay [35]. While a cost analysis was not performed in our study, dalbavancin use has been shown to reduce the cost of hospitalization in general. Certainly, when compared to 4–6 weeks in the hospital receiving parenteral antibiotics (the only alternative for standard of care therapy for patients who do not qualify for OPAT and are unable to be placed in a SNF), treatment with dalbavancin would be expected to decrease the financial burden on hospitals [36]. 

The standard of care for blood stream infections typically involves central venous access, a safe location for antibiotic administration, and prolonged courses of intravenous antibiotics; for the patients in this case series, this was found to be insurmountably difficult to facilitate. In these patients, dalbavancin could potentially be used as salvage therapy. It may also significantly decrease the length of hospital stay due to difficulties in SNF placement, as well as adverse events such as catheter-related infections [34]. However, even with a treatment course that often consisted of only 2 infusions, adherence and follow-up remained an issue in our patient population. Novel interventions in disease treatment will likely be insufficient if there is no clear plan to address barriers to care, such as addiction, in our patients. Complex infections and IVDU are often comorbid conditions and the risks of repeat infection or incomplete treatment are high if we only treat one and not the other. 

## 5. Conclusions

While all healthcare providers should aim to prescribe first-line therapy for any disease state, the ideal treatment is sometimes not feasible. In vulnerable patient populations such as PWID, who are both more likely to develop bacteremia or IE as well as to have barriers to standard treatments, dalbavancin can be a logical alternative. Further study is needed to demonstrate clinical and microbiological non-inferiority of dalbavancin to standard treatments for bacteremia and IE in this group, and to determine the most effective dosing and frequency for a dalbavancin course. 

## Figures and Tables

**Table 1:** Patient Characteristics, Infection Details, Treatment, and Outcomes.

Age (Years); Sex; Race	Active IDU	Infection Type	Organism (s) in Blood Culture	Source	Source Control Achieved	Pre-DAL Antibiotic(s); Total Days	Dalba. Dose(s)	DAL Doses Received/Intended	Intended DAL Course (Days)	Outcome
24; F; Cauc.	Yes	Definitive IE	MRSA, α and γ hemolytic *Streptococci, Bacillus cereus*	Unknown	NA	VAN; 21	1500 mg	1/2	42	LTFU
24; F; Cauc.	Yes	Possible IE	MSSA	Unknown	NA	OXA, CFZ; 21	1500 mg	1/2	42	Cure
27; M; AA	Yes	BSI	*Staphylococcus lugdunensis*	Unknown	NA	VAN; 3	1500 mg	1/2	14	LTFU
29; M; Cauc.	Yes	BSI	MRSA	OM	No	VAN, SAM; 4	1500 mg	1/2	42	Failure
35; M; Cauc.	No	BSI	MSSA, GAS, CoNS	SSTI	NA	VAN, CFZ; 4	1500 mg	1/1	14	Failure (death)
36; M; Cauc.	Yes	Definitive IE	*Streptococcus mitis*	Unknown	NA	CRO, GEN; 11	1500 mg	1/1	14	LTFU
36; M; Cauc.	Yes	BSI	MRSA	PNA/ empyema	Yes	VAN; 30	1500 mg	1/1	42	LTFU
38; M; Cauc.	No	BSI	MRSA	Unknown	NA	VAN; 2	1500 mg; 1000 mg	2/2	28	Cure
38; F; AA	No	BSI	GAS	SSTI	Yes	VAN; 6	1500 mg	1/1	14	Cure
44; M; Cauc.	Yes	Possible IE	MRSA, *Klebsiella pneumonia, Candida albicans* and *tropicalis,*	CLABSI	Yes	VAN, DAP; 27	1500 mg	1/1	42	Failure (death)
50; M; Cauc.	Yes	Dissem. MRSA	sterile	SSTI	Yes	VAN; 13	1500 mg; 1500 mg	2/2	28	Failure
50; M; AA	Yes	BSI	MRSA	SSTI	No	VAN; 6	1500 mg	1/1	14	Cure
50; F; Cauc.	Yes	BSI	MSSA	Unknown	NA	VAN, CFZ; 23	1000 mg; 500 mg	2/2	42	Failure
51; F; Cauc.	Yes	BSI	MSSA	Unknown	NA	VAN, CFZ; 11	1500 mg	1/1	14	Cure
51; M; Cauc.	Yes	BSI	MRSA	OM	Yes	VAN 11	1500 mg; 1000 mg	2/2	28	Cure
55; F; AA	No	BSI	MRSA	CLABSI	Yes	VAN; 8	1500 mg; 1500 mg	2/3	28	Cure
57; M; Cauc.	No	BSI	*Staphylococcus epidermidis, Klebsiella pneumoniae*	Unknown	NA	VAN; 12	1500 mg	1/1	14	Cure
66; M; AA	No	BSI	MRSA, *Enterococcus feacalis Acinetobacter baumanni, Pseudomonas aeruginosa, Candida albicans,*	Unknown	NA	VAN; 8	1500 mg	1/1	14	Failure (death)

AA, African American; Cauc., Caucasian; CFZ, cefazolin; CLABSI, central-line associated blood stream infection; CoNS, coagulase-negative *Staphylococcus*; CRO, ceftriaxone; DAL, dalbavancin; DAP, daptomycin; Dissem., disseminated; F, female; GAS, group A *Streptococcus*; GEN, gentamycin; IDU, injection drug use; IE, infective endocarditis; LTFU, lost to follow-up; M, male; MSSA, methicillin-sensitive *Staphylococcus aureus*; MRSA, methicillin-resistant *Staphylococcus aureus*; NA, not applicable; OM, osteomyelitis; OXA, oxacillin; PNA, pneumonia; SAM, ampicillin–sulbactam; SSTI, skin and soft-tissue infections; VAN, vancomycin.
